# Ameliorative Effect of D-Carvone against Hepatic Ischemia-Reperfusion-Induced Injury in Rats

**DOI:** 10.3390/life12101502

**Published:** 2022-09-27

**Authors:** Maged E. Mohamed, Nancy S. Younis

**Affiliations:** Department of Pharmaceutical Sciences, College of Clinical Pharmacy, King Faisal University, Al-Ahsa 31982, Saudi Arabia

**Keywords:** apoptosis, carvone, hepatic ischemia-reperfusion, HMGB1, inflammation, TLR

## Abstract

Background: D-carvone is a monoterpene that exists in the essential oils of several plant species. Hepatic ischemia-reperfusion (Hep I/R) takes place clinically during different scenarios of liver pathologies. The aim of the current investigation is to disclose the hepato-protective actions of carvone against Hep I/R-induced damage and to reveal the underlying mechanism. Material and methods: Rats were assigned into five groups: sham and carvone plus sham groups, in which rats were administered either saline or carvone orally for three weeks prior to the induction of Hep I/R. In the Hep I/R group, rats were administered saline orally prior to the Hep I/R induction operation. The carvone 25 plus Hep I/R and Carvone 50 plus Hep I/R groups were administered carvone (25 and 50 mg/kg, respectively) for three weeks, followed by the induction of Hep I/R. Results: Liver ischemic animals demonstrated impaired liver function, several histopathological variations, and reduced levels of antioxidant enzyme activities. Furthermore, the Hep I/R groups showed the elevated gene expression of high-mobility group box 1 (HMGB1), toll-like receptors 4 (TLR4), nuclear factor kappa B (NFκB), and LR family pyrin domain containing 3 (NLP3), with subsequent escalated adhesion molecule 1 (ICAM-1), neutrophil infiltration, and several inflammatory mediators, including interleukin 1 beta (IL-1β), interleukin 6 (IL-6), and tumor necrosis factor α (TNF-α), as well as apoptotic markers. Pretreatment with D-carvone alleviated ischemia/reperfusion-induced impaired liver function, diminished the histopathological deviations, and augmented the antioxidant enzymes. In addition, D-carvone mitigated the gene expression of HMGB1, TLR4, NFκB, and NLP3, with a subsequent reduction in ICAM-1, neutrophils infiltration, inflammatory mediators, and apoptotic markers. Conclusion: Rats pretreated with D-carvone exhibited hepato-protective actions against Hep I/R-induced damage via the downregulation of HMGB1, TLR4, NFκB, NLP3, associated inflammatory mediators, and apoptotic markers.

## 1. Introduction

Hepatic ischemia-reperfusion (Hep I/R) takes place clinically during different situations of liver pathologies, such as following liver transplantation, liver resection, or hemorrhagic shock, which leads to high morbidity and mortality [1]. The early phase of Hep I/R injury is characterized by Kupffer cell-induced oxidative stress, resulting in hepatocellular injury followed by a massive neutrophil infiltration [2]. Injured hepatic cells release damage-associated molecular patterns (DAMP), one of which is the high-mobility group box 1 (HMGB1), a ubiquitous nuclear and cytosolic protein that is passively released by ischemia or cell injury in the absence of an invasion [3]. HMGB1 interacts with several receptors, which transduces activation signals from exogenous toll-like receptors (TLRs) and endogenous receptor for advanced glycation endproducts (RAGE) ligands [4]. HMGB1 activates immune cells via pattern recognition receptors, including TLRs (TLR2, TLR4, and TLR9) and RAGE [5]. Hep I/R injury prompts innate immunity by triggering TLRs in both parenchymal and nonparenchymal hepatocytes [6]. TLR4 engagement on actively phagocytic nonparenchymal cells such as Kupffer cells is detrimental in liver I/R-induced injury and inflammation [7]. Upon the interaction of HMGB1with TLRs, the inflammatory response is induced by the production of proinflammatory mediators (e.g., TNF-α, IL-1β, and IL-6) through the activation of the myeloid differentiation primary response 88 (MyD88)-dependent NF-κB pathway. Furthermore, the characteristics of Hep I/R include hepatocyte death, the release of DAMPs, inflammatory cell infiltration, Kupffer cell activation, ROS production, and disruption of hepatic sinusoidal endothelial cells (LSEC), which can all lead to inflammasome activation [8]. Nucleotide-binding and oligomerization domain (NOD)-like receptors (NLRs) play a role in the formation of inflammasomes. Inflammasomes are intracellular multiprotein complexes expressed in both the parenchymal and non-parenchymal cells of the liver in response to cellular danger signals to activate caspase-1 and release different ILs such as IL-1β and IL-18, among other inflammatory mediators [8].

Owing to the contribution of oxidative stress and inflammatory responses in the pathogenesis of Hep I/R, prominence was specified to plant-derived antioxidant compounds as possible solutions. Carvone, of the Lamiaceae and Asteraceae families, is a natural monocyclic monoterpene enclosed in several aromatic plants’ essential oils [9]. Carvone has two stereochemical isomers (enantiomers): L-carvone (or R-(−)-carvone) and D-carvone (or S-(+)-carvone) [9]. L-carvone has a spearmint-like odor, and it is the main component in the mint family’s (Lamiaceae) essential oils, particularly spearmint [10]. D-carvone has a strong, caraway-like aroma, and it is the principal constituent of caraway seeds’ (Carum carvi) essential oils. It also occurs in dill (Anethum graveolens) seed oil [11]. Several beneficial effects of D-carvone have been verified. For instance, D-carvone modulated 1, 2-dimethylhydrazine (DMH)-induced pre-neoplastic lesions, oxidative stress, and biotransforming enzymes in an experimental model of rat colon carcinogenesis, offering an effective chemo-preventive compound against colon carcinogenesis [12]. D-carvone restrained high-fat, diet-induced obesity, hepatic steatosis, and insulin resistance [13], and it ameliorated hyperglycemia via regulating the key enzyme activities of carbohydrate metabolism in STZ-induced diabetic animals [14]. D-Carvone exhibited neuroprotective actions via constraining the cerebral ischemia/reperfusion-induced inflammatory response via deterring the TLR4/NLRP3 signaling pathway. Furthermore, D-carvone showed cardioprotective effects against doxorubicin (DOX)-induced cardiotoxicity and potentiated the anticancer effect of DOX [15]. D-carvone attenuated CCl4-induced liver fibrosis by deterring oxidative stress and the TGF-ß 1/SMAD3 pathway, which presented the compound as a possible candidate for inhibiting liver fibrosis, as well as other oxidative stress-related hepatic diseases [16]. Carvone suppressed oxidative stress and inflammation in the liver of immobilized rats [17]. Several studies have revealed that D-carvone exhibits anti-fibrotic and antioxidant [16], anti-inflammatory [17], anti-arthritic [18], anticancer [19], antinociceptive [20], and anti-ulcerative colitis [21] effects. In spite of all the revealed pharmacological actions of carvone, the compound’s action against Hep I/R- induced insult has not been explored before. Accordingly, in the current study, we tried to disclose the hepato-protective actions of D-carvone against Hep I/R damage. Furthermore, we sought to reveal the possible underlying mechanisms that account for this protection via exploring D-carvone’s effects on the HMGB1, TLR4, NFκB, NLP3 pathways and the associated inflammatory mediators and apoptotic markers.

## 2. Materials and Methods

### 2.1. Animal Acquisition and Ethical Approval

Wistar male rats (200–230 g) were acquired from the Experimental Animal Research Centre, King Saud University, Riyadh, KSA. The animals were maintained with standard laboratory food and water ad libitum in ventilated cages system (12 h light/dark cycles, 20–23 °C) one week before the experiment and throughout the whole experiment. The Institutional Animal Care and Use Committee of King Faisal University allowed the experimental protocol (KFU-REC-2022-FEB-EA000409). All the experiments were accomplished in agreement with the relevant procedures and regulations of the Ethical Conduct for the Use of Animals in Research at King Faisal University.

### 2.2. Hep I/R Injury Procedure 

Hep I/R surgery was implemented, as mentioned before [22,23]. In brief, the rats were anesthetized using isoflurane with oxygen (2%, 0.5 L/h) and positioned in the supine position. In the animals’ upper abdomen, a midline opening was executed to expose the hepatic portal. The branches of the left and middle portal veins and hepatic arteries were clipped using a noninvasive vascular clamp. The liver’s lobe color in the ischemic area changed from deep red to pale white, validating effective liver ischemia initiation. After 1 h, the clamp was removed, permitting the hepatic blood current reperfusion phase for 24 h. In the sham-operated animals, the midline openings were executed to expose the branches of the left and middle portal veins and hepatic arteries; however, blocking the blood flow was not permitted.

### 2.3. Experimental Design and Sample Obtainment 

D-carvone, (S-(+)-carvone or p-Mentha-6,8-dien-2-one, C10H14O, molecular weight: 150.22, product number 435759) was purchased from Merck, Darmstadt, Germany. The rats were assigned randomly into five groups (n = 6). The sham and carvone plus sham rat groups were administered either saline or carvone (50 mg/kg), respectively, [16,17] orally via gavage once daily for three weeks before the sham procedure. The Hep I/R group was given saline orally once daily for three weeks prior to the induction of Hep I/R. The carvone 25 plus Hep I/R and carvone 50 plus Hep I/R groups were administered carvone orally (25 and 50 mg/kg, respectively) once daily for three weeks, followed by the induction of Hep I/R. 

### 2.4. Assessment of Liver Function Parameters

At the end of the reperfusion phase, blood samples were harvested from the abdominal aorta of the anesthetized animals, and the sera were isolated for the measurement of alanine aminotransferase (ALT, ab105134/K752-100), aspartate aminotransferase (AST, ab263883), alkaline phosphatase (ALP, ab83369), and lactate dehydrogenase (LDH, ab102526) using diagnostic kits obtained from Abcam (Boston, MA, USA). 

### 2.5. Assessment of Parameters in Liver Tissues

The hepatic tissue was collected and divided into four portions. The first portion was homogenized in ice-cold saline and stored at −20 °C for the biochemical and ELISA assays. The second hepatic portion was mixed with lysis buffer for the Western blot analysis, whereas the third part was conserved in 10% formalin for the histopathological and immunohistochemical (IHC) investigations. The last portion was mixed in RNA later solution and kept at −20 °C for gene expression assay via quantitative real-time polymerase chain reaction (qRT-PCR). 

#### 2.5.1. Histopathological and Immunohistochemistry Investigations 

After fixing the ischemic liver fractions with 10% formalin, the fractions were embedded in paraffin, cut into 5 µm thick paraffin slices, dewaxed with xylene twice, fully hydrated with ethanol, and rinsed with tap water. The ischemic hepatic sections were stained with hematoxylin and eosin (H&E). The hepatic injuries were evaluated under a light microscope by two independent pathologists blinded to the experimental groups. The hepatic ischemic-induced injuries were graded as follows: grade 0 represented minimal or no evidence of injury; grade 1 represented mild injury described by cytoplasmic vacuolization and focal nuclear pyknosis; grade 2 signified moderate damage demonstrating cytoplasmic vacuolization but no frank necrosis, sinusoidal dilatation, or congestion; grade 3 denoted moderate to severe damage associated with necrosis, cytoplasmic hypereosinophilia, extensive sinusoidal dilatation, and congestion; and grade 4 indicated severe injury involving severe necrosis and hemorrhage into the liver chords causing the loss of hepatic construction [24]. 

For the immunohistochemistry (IHC) procedure, the expression of TLR4 and NFκB p65 were examined as revealed previously [25]. TLR4 and NFκB p65 antibodies (1: 1000, Catalog # 48-2300, 14-6731-81, respectively) were obtained from Thermo Fisher Scientific, Cambridge, UK. NIS-Elements software was used for the quantitative analysis of TLR4 and NFκB. First, the area of the immunohistochemical reaction in the picture was selected. Then, the average optical density in the selected area of each picture was measured. Positive cells were counted under 400× magnification, observing 10 consecutive, non-overlapping fields per animal in a blinded manner to produce the scoring scale.

#### 2.5.2. Assessment of Hepatic Oxidative Stress Status 

Malondialdehyde (MDA; ab238537), glutathione peroxidase (GSH-Px; ab102530), and glutathione reductase (GSH-R; ab83461) ELISA kits were acquired from Abcam Inc. (Cambridge, UK). Superoxide dismutase (SOD; MBS036924) and catalase (MBS726781) ELISA kits were obtained from My BioSource (San Diego, CA, USA). All the procedures were performed in agreement with the manufacturer’s directions. 

#### 2.5.3. Gene Expression Experiments (Real-Time PCR) 

Real-time PCR was performed according to the technique described elsewhere [24]. Briefly, the Trizol reagent kit (Invitrogen, Waltham, MA, USA) and reverse transcription-polymerase chain reaction (RT-PCR) kit (TaKaRa (Shiga, Japan), Cat. No. RR037A) were used to cleanse the total RNA and inverse transcription reactions, respectively. In total, 20 μL of the reaction volume was mixed with 1 μL total RNA (1 μg/μL), which was incubated at 42 °C for 15 min, followed by 95 °C for 2 min, and the generated cDNA was stored at −20 °C. In total, 50 μL of the PCR reaction mixture comprised × 50 ROX Reference Dye (1 μL), sense and antisense primers (1 μL each, with the specific primers mentioned in Table 1), × 2 SYBR Green PCR Master Mix (25 μL), cDNA template (4 μL), and sterilized distilled H2O (18 μL). The PCR reaction condition incorporated pre-denaturing at 95 °C for 10 s, and then 40 cycles of 95 °C/5 s, 60 °C/30 s, and 72 °C/1 min. Quantification analyses were completed via an Opticon-2 Real-Time PCR reactor (MJ Research, Reno, NV, USA). A Step PE Applied Biosystems (Perkin Elmer, Waltham, MA, USA) was used to analyze the real-time PCR results. Expression of the target gene was measured and correlated to the reference gene (β-actin). β-actin expression was used for sample normalization, where the 2−ΔΔCT equation was used for relative expression determination.

#### 2.5.4. Determination of Inflammation and Apoptotic Signaling Markers

Inflammation markers including TNF-α (ab46070), IL-1β (ab100768), IL-6 (ab100772), IL-10 (ab133112), and ICAM-1 (CD54) (ab100763) ELISA kits were obtained from Abcam Co., Eugene, OR, USA. As for the apoptotic signaling markers, cleaved caspase-3 (KHO1091) was purchased from Thermo Fisher Scientific Inc. Waltham, MA, USA, and caspase-9 (LS-F4141) was acquired from Biocompare, San Francisco, CA, USA. These markers were measured according to the manufacturer’s instructions using a SpectraMax i3X (Molecular devices San Jose, CA, USA) microplate reader. 

### 2.6. Statistical Analysis

The data are presented as means ± SDs. For multiple comparisons, one-way ANOVA followed by a Tukey–Kramer post hoc test were performed. A 0.05 level of probability was used as the significance level. All statistical analyses were performed using Graph Pad software (version 5, San Diego, CA, USA).

## 3. Results

### 3.1. D-Carvone Pretreatment Improved Hepatic Function Tests in Hep I/R 

**Figure 1:**
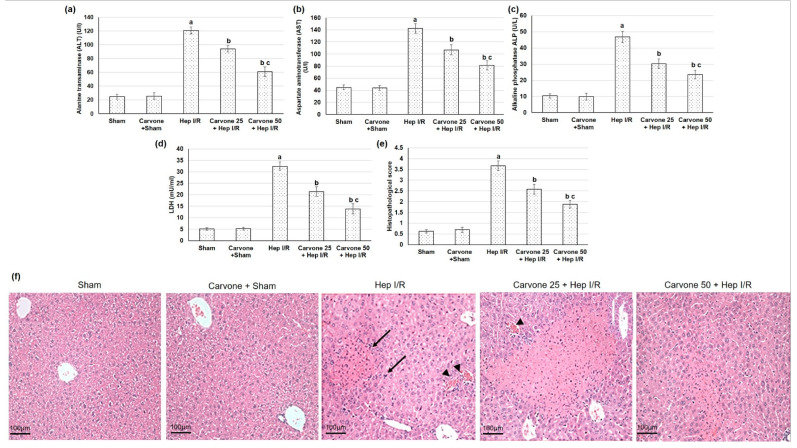
Effects of D-carvone (25 and 50 mg/kg) administration for 3 weeks prior to hepatic ischemia/perfusion (Hep I/R) in the hepatic function tests, including (**a**) ALT, (**b**) AST, (**c**) ALP, and (**d**) LDH, and (**e**) on the histopathological scoring scale and (**f**) in the histopathological hepatic sections stained with hematoxylin and eosin (H&E), in which the sham group showed minimal or no evidence of injury, while the Hep I/R groups showed severe confluent coagulative necrosis (arrow), disintegration, and hemorrhage (arrowhead) leading to the loss of hepatic architecture structure. All the values are stated as means ± SDs, with n = 6. **a**: designated as statistically significant compared to the sham group; **b**: designated as statistically significant compared to the H I/R group; **c**: designated as statistically significant compared to the carvone 25 plus Hep I/R group (*p* < 0.05) using one-way ANOVA followed by a Tukey’s post hoc test.

The liver ischemic animals presented with impaired liver function, as shown by the elevated serum levels of ALT, AST, ALP, and LDH (*p* < 0.05) when compared to the sham animals. Alternatively, the D-carvone pretreatment alleviated the ischemia/reperfusion-induced impaired liver function, as established by the decrease in the serum levels of ALT, AST, ALP, and LDH (*p* < 0.05). Treatment with D-carvone (25 and 50 mg/kg) resulted in percentage decreases in ALT of 22% and 49.6%, in AST of 24.7% and 42.90%, in ALP of 35.25% and 49.5%, and in LDH of 43.03% and 57.31%, respectively, as illustrated in Figure 1a–d. 

### 3.2. Histopathological Examination 

The sham-experienced animals displayed regular histology architectures, whereas the hepatic ischemic reperfused animals exhibited several histopathological variations, including diffuse necrosis with disorganized parenchyma and dilated sinusoids (Figure 1e,f). Alternatively, the D-carvone pretreated groups presented reconstructed parenchyma cells, fewer hepatocellular necrosis, dropped congested hepatic sinusoids, and portal vasculatures with slight vacuolation, as shown in Figure 1e,f. Furthermore, the histopathological scoring was extensively amplified in the ischemic reperfused animals, whereas carvone demonstrated a substantial reduction in the histopathological scoring system when related to the hepatic ischemic animals, as illustrated in Figure 1e.

### 3.3. D-Carvone Pretreatment Reduced HMGB1/TLR4/NFκB/NLP3 Signaling in Hep I/R 

**Figure 2:**
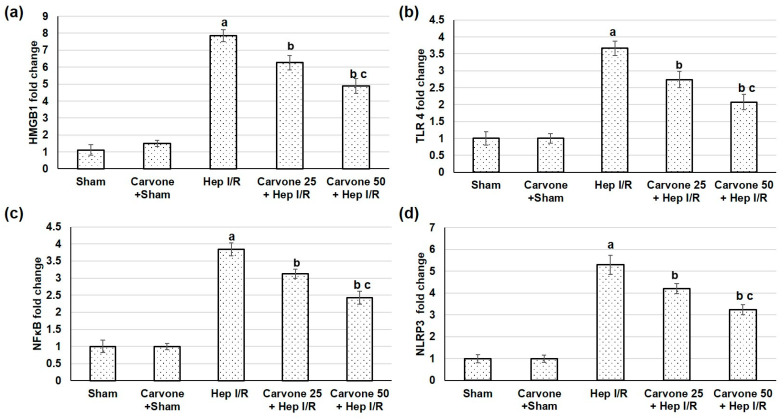
Effects of D-carvone (25 and 50 mg/kg) administration for 3 weeks prior to hepatic ischemia/perfusion (Hep I/R) on the gene (mRNA) expression levels of (**a**) HMGB1, (**b**)TLR4, (**c**) NFKB, and (**d**) NLRP3, respectively, in the Hep I/R-induced injury groups. All values are stated as means ± SDs, with n = 5. **a**: designated as statistically significant compared to the sham group; **b**: designated as statistically significant compared to the H I/R group; **c**: designated as statistically significant compared to the carvone 25 plus Hep I/R group (*p* < 0.05) using one-way ANOVA followed by a Tukey’s post hoc test.

**Figure 3:**
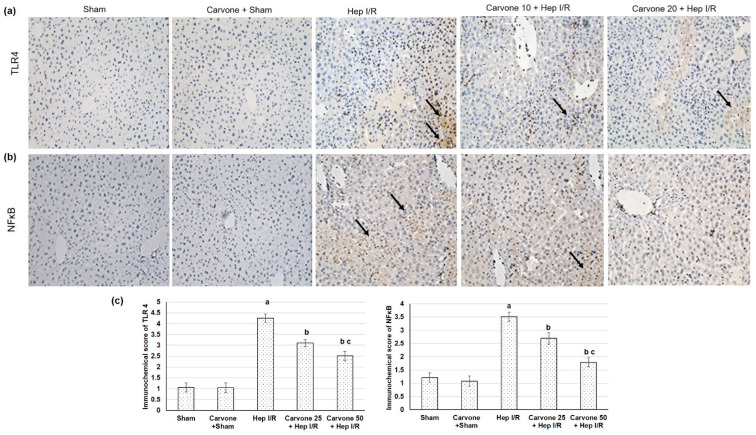
Effects of D-carvone (25 and 50 mg/kg) administration for 3 weeks prior to hepatic ischemia/perfusion (Hep I/R) on the hepatic immunohistochemical assay of (**a**) TLR4, (**b**) NFκB, (**c**) TLR4, and NFκB scoring scales in rats with H I/R-induced injury. The sham group showed negative immunoreactivity in the scattered cells, while the Hep I/R groups showed strong immunoreactivity in the scattered cells (arrow). The carvone 10 plus Hep I/R and carvone 20 plus Hep I/R groups showed mild immunoreactivity in the scattered cells (arrow). All the values are stated as means ± SDs. **a**: designated as statistically significant compared to the sham group; **b**: designated as statistically significant compared to the H I/R group; **c**: designated as statistically significant compared to the carvone 25 plus Hep I/R group (*p* < 0.05) using one-way ANOVA followed by a Tukey’s post hoc test.

The pathway we focused on in the current study was the HMGB1/TLR4/NFκB/NLP3 signaling pathway; therefore, we studied the gene expression of HMGB1, TLR4, NFκB, and NLP3. The liver ischemic reperfused animals demonstrated increased gene expressions of HMGB1, TLR4, NFκB, and NLP3, as shown in Figure 2, suggesting amplified HMGB1/TLR4/NFκB/NLP3 pathways due to the ischemia reperfusion-induced injuries. However, when D-carvone preconditioning was performed earlier than the liver ischemia-reperfusion insult, it resulted in mitigated HMGB1/TLR4/NFκB/NLP3 signaling, as established by the lowered gene expressions of HMGB1, TLR4, NFκB, and NLP3. Furthermore, IHC analysis exposed that TLR4 and NFκB (Figure 3) sparingly appeared in the sham-experienced animals, whereas the Hep I/R groups intensely demonstrated TLR4 and NFκB positive cell manifestations (*p* < 0.05). In the D-carvone pretreated groups, TLR4 and NFκB positive cells declined compared with the Hep I/R groups (*p* < 0.05). These outcomes suggest that D-carvone may exhibit a hepatoprotective action via inhibiting the HMGB1/TLR4/NFκB/NLRP3 pathway.

### 3.4. D-Carvone Pretreatment Improved Anti-Oxidant Capacity in Hep I/R

**Figure 4:**
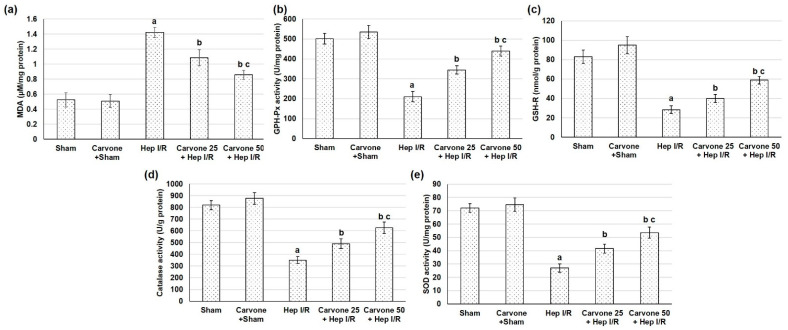
Effects of D-carvone (25 and 50 mg/kg) administration for 3 weeks prior to Hep I/R on lipid peroxidation in (**a**) MDA content and on antioxidant enzymes activities, including (**b**) glutathione peroxidase (GSH-Px), (**c**) glutathione reductase (GSH-R), (**d**) catalase, and (**e**) superoxide dismutase (SOD). All the values are stated as means ± SDs, with n = 6. **a**: designated as statistically significant compared to the sham group; **b**: designated as statistically significant compared to the H I/R group; **c**: designated as statistically significant compared to the carvone 25 plus Hep I/R group (*p* < 0.05) using one-way ANOVA followed by a Tukey’s post hoc test.

The hepatic I/R samples displayed reduced levels of antioxidant enzyme activities, including SOD, catalase, GSH-Px, and GSH-R, while D-carvone administration prior to Hep I/R resulted in augmented activities of these antioxidant enzymes in hepatic tissue samples when related to the Hep I/R group (Figure 4). Regarding lipid peroxidation, the Hep I/R animals presented significantly superior levels of MDA content, indicating lipid peroxidation augmentation. Such an effect was markedly lower in the animals pretreated with D-carvone (25 and 50 mg/kg) before Hep I/R. 

### 3.5. D-Carvone Pretreatment Reduced Neutrophils Infiltration and Inflammation Associated with Hep I/R

**Figure 5:**
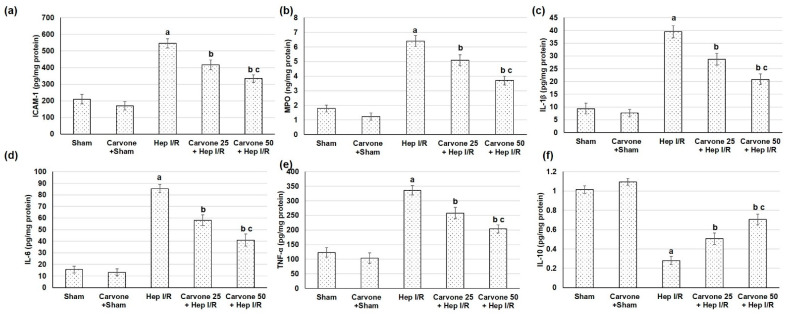
Effects of D-carvone (25 and 50 mg/kg) administration for 3 weeks prior to hepatic ischemia/perfusion (Hep I/R) on the levels of (**a**) ICAM-1, (**b**) MPO, (**c**) IL-1β, (**d**) IL-6, (**e**) TNF-α, and (**f**) IL-10 in animals with Hep I/R-induced injury. All the values are stated as means ± SDs, with n = 6. **a**: designated as statistically significant compared to the sham group; **b**: designated as statistically significant compared to the H I/R group; **c**: designated as statistically significant compared to the carvone 25 plus Hep I/R group (*p* < 0.05) using one-way ANOVA followed by a Tukey’s post hoc test.

Intercellular adhesion molecule 1 (ICAM-1) with subsequent neutrophil infiltration, as signified by MPO levels, were significantly prompted in the Hep I/R animals (Figure 5a,b). Yet, with D-carvone administration, the adhesion molecule ICAM-1 and neutrophil infiltration (MPO) were downregulated. The inflammatory mediators, including IL-1β, IL-6, and TNF-α, were escalated in the animals that experienced Hep I/R compared to the sham animals. IL-10 was diminished as a result of Hep I/R induction. These inflammatory mediator intensifications were weakened in animals pretreated with D-carvone (25 and 50 mg/kg) and, on the contrary, IL-10 was elevated (Figure 5c–f).

### 3.6. D-Carvone Pretreatment Reduced the Apoptosis Response Associated with Hep I/R

**Figure 6:**
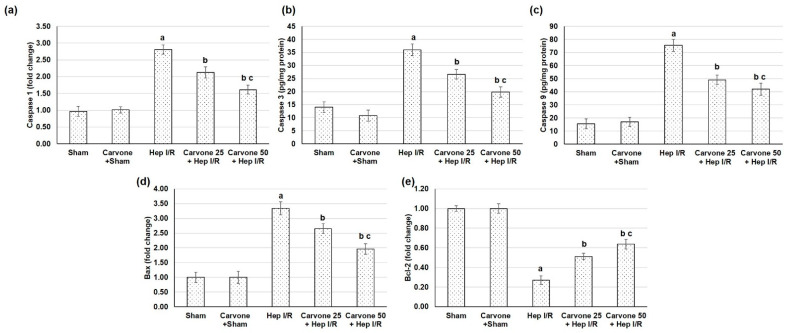
Effects of D-carvone (25 and 50 mg/kg) administration for 3 weeks prior to hepatic ischemia/perfusion (Hep I/R) on (**a**) caspase 1, (**b**) caspase 1, and (**c**) caspase 9 and on the gene expression levels of (**d**) Bax and (**e**) Bcl2 in Hep I/R-induced apoptosis. All the values are stated as means ± SDs, with n = 6. **a**: designated as statistically significant compared to the sham group; **b**: designated as statistically significant compared to the H I/R group; **c**: designated as statistically significant compared to the carvone 25 plus Hep I/R group (*p* < 0.05) using one-way ANOVA followed by a Tukey’s post hoc test.

The groups with experimental Hep I/R surgery exhibited exaggerated apoptotic markers comprised of caspase 1, 3, and 9 levels and Bax gene expression, while Bcl2 gene expression was reduced, as shown in Figure 6. On the other hand, the administration of D-carvone resulted in apoptosis mitigation, as demonstrated by the lowered caspase 1, 3, and 9 and Bax, with elevated Bcl2 expression, confirming the anti-apoptotic effect of the compound.

## 4. Discussion

D-carvone is a monoterpene that exists in the essential oils of several plant genera, including Mentha, Origanum, Rosmarinus, Thymus, and others [9]. D-carvone has shown several medical actions, for instance, it has exhibited anticancer actions in myeloma cells, mediated through the p38 MAPK signaling pathway [19]. Furthermore, D-carvone decreased peripheral nerve excitability, ensuring antinociceptive activity [20]. During Hep I/R-induced damage, hepatocyte death causes the release of several DAMPs, infiltration of numerous inflammatory cells, and production of ROS, which may all lead to further hepatic injury [8]. In the current study, ischemic reperfused animals displayed impaired liver function as revealed by the elevated ALT, AST, ALP, and LDH levels, and they exhibited numerous histopathological variations when compared to the sham animals. These results coincide with other studies [26,27,28],whereas D-carvone pretreatment alleviated ischemia/reperfusion-induced impaired liver function and minimized the histopathological deviations. Similarly, D-carvone treatment lowered the levels of liver function enzymes in immobilized rats [17], as well as that associated with CCl4-induced liver fibrosis [16] and STZ-induced hepatotoxicity [14]. 

Oxidative stress plays an imperative role in Hep I/R-induced injury. Excessive levels of reactive oxygen species (ROS) can cause tissue damage and cell death by binding and fluctuating numerous cellular macromolecules (including DNA, proteins, and lipids), subsequently affecting their functions [29]. Some studies have also suggested that ROS contribute to inflammasome activation [8]. Furthermore, myeloperoxidase has been shown to be amplified in ischemic hepatic lobes and was significantly lower in Hep I/R-injured TLR4 mutant mice [7]. In the existing study, the Hep I/R samples displayed a reduced level of antioxidant enzyme activities and elevated lipid peroxidation, while D-carvone administration prior to Hep I/R resulted in augmented antioxidant enzyme activities and markedly lowered MDA levels in hepatic tissue samples. Previously, D-carvone has been shown to have exceptional antioxidant activity as it significantly enhanced oxidant/antioxidant status and lowered lipid peroxidation levels in CCl4-induced liver fibrosis animals [16], arthritic rats [18], and immobilized rats [17], among other models. 

The results of the existing study showed that the ischemic reperfused animals exhibited an increase in the gene expression of HMGB1, TLR4, NFκB, and NLP3, suggesting amplified HMGB1/TLR4/NFκB/NLP3 pathways associated with ischemia reperfusion-induced damage. Previous reports have shown the detrimental roles of these pathways [26,30,31]. HMGB1 is released as an early mediator of ischemia-reperfusion [5]. HMGB1 release occurs during programmed cell death from at least two sources: directly from the apoptotic cells and by the activated monocytes, which secrete HMGB1 following exposure to apoptotic cell bodies [32]. TLR4 is a receptor of HMGB1 in mediating macrophage activation, cytokine release, and tissue injury [33,34]. This signaling activates IKB kinase (IKK)-β, IKK-α, and the nuclear translocation of activated NF-κB [4]. Several studies have explored the role of HMGB1/TLR4 pathway intensification in hepatic ischemia-induced injury [35,36,37]. NLRPs are subfamilies of NOD-like receptors, members of the pattern recognition receptor family, which play a role in the recognition of ligands [8]. NLRP3 expression is firmly regulated at the transcriptional level via NF-κB [38]. NLRP3 activation requires two signals: the first is cell priming with an NF-κB activator, such as the TLR4-ligand LPS, which is the first step of NLRP3 inflammasome activation leading to the upregulation of NLRP3 expression, and the second includes a broad variety of activators, including reactive oxygen species (ROS) [8]. Silencing NLRP3 ameliorated I/R-induced hepatocellular injury and reduced IL-1β, IL-18, IL-6, and TNF-α release via the inhibition of caspase-1 and NF-κB activity [39]. Thus, the NLRP3 inflammasome and TLR4 signal work together to regulate inflammation [40]. Altogether, Hep I/R activates innate immunity with subsequent cytokine release, which directly mediates the development of systemic inflammatory responses and hepatic tissue injury. 

On the other hand, performing D-carvone administration earlier than Hep I/R insult results in the mitigation of HMGB1/TLR4/NFκB/NLP3 signaling, as established by the lowered gene expression of HMGB1, TLR4, NFκB, and NLP3. An earlier study showed that D-carvone suppresses the NLRP3 inflammasome-induced inflammation in cerebral ischemia/reperfusion. D-carvone decreased the gene expression of NLRP3, caspase-1, TNF-α, ASC, IL-1β, and TLR4, which are known NLR3 inflammasome components [14]. 

The outcomes of the present investigation showed that Hep I/R significantly prompted the release of intercellular adhesion molecule 1 (ICAM-1) with subsequent neutrophil infiltration. One of the proposed mechanisms to mitigate hepatic ischemia injury is to deter inflammatory mediator-induced damage, as demonstrated in earlier studies [22,41,42]. Yet, with D-carvone administration, the adhesion molecule ICAM-1 and neutrophil infiltration (MPO) were downregulated. The inflammatory mediators including IL-1β, IL-6, and TNF-α were escalated in the animals which experienced Hep I/R compared to the sham animals. These inflammatory mediator (IL-1β, IL-6, and TNF-α) intensifications were weakened in the animals who were administered D-carvone (25 and 50 mg/kg). D-carvone showed an anti-inflammatory effect in several models. For instance, D-carvone protected against dextran sulfate sodium-induced ulcerative colitis in Balb/c mice and LPS-induced RAW cells via the inhibition of COX-2 and TNF-α [21]. Likewise, D-carvone presented anti-arthritic activity against complete Freund’s adjuvant-induced arthritis in rats via significantly modulating the levels of inflammatory cytokines (IL-6, IL-1β, and TNF-α) [18]. Further, D-carvone is a potential anti-allergic drug as it inhibited leukocyte infiltration and mucus production in the lungs, which was correlated with the decreased production of ovalbumin (OVA)-specific IgE and increased concentrations of IL-10 [43]. Similarly, D-carvone lowered cytokine production in brain ischemia-reperfusion [14]. Additionally, carvone inhibited leukocyte infiltration and mucus production in the lungs, which was correlated with the decreased production of OVA-specific IgE and increased concentrations of IL-10 in bronchoalveolar lavage (BAL) [43]. As for hepatic actions, D-carvone lowered TNF-α, IL-1β, IL-6, and NF-κB gene expression and reduced the infiltration of inflammatory cells in the liver parenchyma in the livers of immobilized rats [17]. Consistent with the role of TNF-α in hepatic I/R, serum TNF-α was diminished in TLR4 mutant mice subjected to hepatic I/R injury, demonstrating the important role of TLR4 via TNF- α in hepatic I/R injury [7]. 

Furthermore, the groups that experienced Hep I/R surgery exhibited exaggerated apoptotic markers comprised of caspase 1, 3, and 9 levels and Bax gene expression, while Bcl2 gene expression was reduced. On the other hand, the administration of D-carvone resulted in apoptosis mitigation as demonstrated by the lowered caspase 1, 3, and 9 levels and Bax and elevated Bcl2 expression. This confirms the anti-apoptotic effect of D-carvone, which is likely due to improved antioxidant activity and diminished inflammation in the hepatic tissue. 

## 5. Conclusions

Pretreatment with D-carvone alleviated ischemia/reperfusion-induced impaired liver function, minimized histopathological deviations, and augmented antioxidant enzymes. In addition, D-carvone treatment mitigated the gene expression of HMGB1, TLR4, NFκB, and NLP3, with subsequent reductions in ICAM-1, neutrophils infiltration, inflammatory mediators, and apoptotic markers. D-carvone exhibited hepato-protective actions against Hep I/R-induced damage via the downregulation of the HMGB1/TLR4/NFκB/NLP3 pathways, associated inflammatory mediators, and apoptotic markers. These data could suggest utilizing D-carvone pretreatment as a potential new therapeutic approach for protection against hepatic I/R in patients expected to experience Hep I/R. 

## Figures and Tables

**Table 1:** Primer sequences used in the real-time PCR experiments.

Markers	Accession Number (Genbank)	Primer Sequence (5′ to 3′)	
		Forward Primer	Reverse Primers
HMGB-1	XM_039100270	5′-AGGCTGACAAGGCTCGTTATG-3′	3′-TGTCATCCGCAGCAGTGTTG-5′
TLR4	NM_019178	5′-GCTGCCAACATCATCCAGGAAGG-3′	3′-TGATGCCAGAGCGGCTACTCAG-5
NFκB	L26267.1	5′-TGGGACGACACCTCTACACA-3′	3′-GGAGCTCATCTCATAGTTGTCC-5′
NLRP3	NM_00111642.1	5′-CAGACCTCCAAGACCACGACTG-3′	3′-CATCCGCAGCCAATGAACAGAG-5′
Caspase 1	NM_012762.3	5′-TGCCTGGTCTTGTGACTTGGAG-3′	3′-ATGTCCTGGGAAGAGGTAGAAACG-3′
Bcl-2	NM_016993.1	5′-CCGGGAGATCGTGATGAAGT-3′	3′-ATCCCAGCCTCCGTTATCCT-5′
Bax	NM_017059.2	5′-GTGGTTGCCCTCTTCTACTTTG-3′	3′-CACAAAGATGGTCACTGTCTGC-5′
β-actin	NM_0 3144.3	5′-TGACAGGATGCAGAAGGAGA-3′	3′-TAGAGCCACCAATCCACACA-5′

## Data Availability

Not applicable.
